# Sex-Specific Transcriptome Differences in Substantia Nigra Tissue: A Meta-Analysis of Parkinson’s Disease Data

**DOI:** 10.3390/genes9060275

**Published:** 2018-05-25

**Authors:** Elisa Mariani, Lorenza Lombardini, Federica Facchin, Fabrizio Pizzetti, Flavia Frabetti, Andrea Tarozzi, Raffaella Casadei

**Affiliations:** 1 Department for Life Quality Studies, University of Bologna, 47900 Rimini, Italy; elisa.mariani11@unibo.it (E.M.); lorenza.lombardini2@unibo.it (L.L.); andrea.tarozzi@unibo.it (A.T.); 2 Department of Experimental, Diagnostic and Specialty Medicine, University of Bologna, 40126 Bologna, Italy; federica.facchin2@unibo.it (F.F.); fabrizio.pizzetti2@unibo.it (F.P.); flavia.frabetti@unibo.it (F.F.)

**Keywords:** Parkinson’s disease, gene expression, sex, substantia nigra, microarray

## Abstract

Parkinson’s disease (PD) is one of the most common progressive neurodegenerative diseases. Clinical and epidemiological studies indicate that sex differences, as well as genetic components and ageing, can influence the prevalence, age at onset and symptomatology of PD. This study undertook a systematic meta-analysis of substantia nigra microarray data using the Transcriptome Mapper (TRAM) software to integrate and normalize a total of 10 suitable datasets from multiple sources. Four different analyses were performed according to default parameters, to better define the segments differentially expressed between PD patients and healthy controls, when comparing men and women data sets. The results suggest a possible regulation of specific sex-biased systems in PD susceptibility. TRAM software allowed us to highlight the different activation of some genomic regions and loci involved in molecular pathways related to neurodegeneration and neuroinflammatory mechanisms.

## 1. Introduction

Parkinson’s disease (PD) is a common progressive neurodegenerative disease, affecting the older population (1–2% prevalence in individuals over 65 years, about 4% over the age of 85 years) [1,2]. The main clinical manifestations of the disease include resting tremor, bradykinesia (slowed movements), rigidity (increased muscular tone), postural instability, and gait impairment. Besides motor symptoms, PD patients manifest a wide range of non-motor symptoms such as depression, fatigue, sleep disturbance and autonomic dysfunction [3,4]. It has been demonstrated that the clinical manifestations of the disease, in particular motor symptoms, are mainly a consequence of the neurodegeneration of dopaminergic (DA) neurons in the substantia nigra (SN) pars compacta [5], and that symptoms usually appear when about 50–60% of these neurons are lost [2,6]. Furthermore, another pathological hallmark of PD is the presence of eosinophilic intraneuronal cytoplasmic protein-rich inclusions known as Lewy bodies (LBs) [7].

Parkinson’s disease is considered a complex disease with an etiology that, to date, is not completely understood. Several factors, including genetic susceptibility and environmental influences, have been identified as possible causes. It has been demonstrated that Mendelian form of PD are caused by autosomal dominant and recessive gene mutations; likewise, genome wide experiments have highlighted an increasing number of loci that can be predisposed to the development of the sporadic form of the disease [8,9,10,11,12].

In addition to the well-known Mendelian transmission of PD, several studies have recently drawn attention to the role of epigenetics in the neurodegeneration that occurs in PD patients [11,13,14]. Epigenetic mechanisms in PD involve DNA demethylation, over-expression of the *SNCA* (synuclein α) gene and deregulation of the histone acetylation/deacetylation equilibrium [15,16,17,18]. Furthermore, the ubiquitin-proteasome system (UPS) has a remarkable impact in inherited PD through the well-known parkin protein, an E3 ubiquitin-ligase protein that changes in PD, loses its function and leads to an accumulation of anomalous proteins [19]. Finally, long non-coding RNA (lncRNA) and microRNA (miRNA) are thought to be involved in neuronal pathways and diseases such as PD [20].

As well as the genetic component, ageing [21] and sex [22,23] can also contribute as risk factors to the onset of the disease. While the role of ageing in PD has already been long established, epidemiological studies have also shown that, regardless of age and ethnicity, the male sex is associated with a higher development rate of PD. In fact, on average, men are twice as likely to develop PD and the disease is 1.3–3.7 times more prevalent in men than in women [22,23,24,25,26,27]. Clinical studies have demonstrated that sex differences can also influence the age of PD’s onset and its symptomatology. Indeed, women develop PD almost two years later than men and initially display tremor symptoms instead of bradykinesia or rigidity and more pronounced symptoms such as depression, anxiety, pain, and sleep disturbances [22,24,27].

It has been hypothesized that estrogens may have an important role in defining such differences; several experiments have shown that estrogens could have a neuroprotective activity towards the dopaminergic (DA) system, both at dopamine synthesis and function level [22,23,24,27,28,29]. It has also recently been suggested that basic sex differences in brain organization, structure and function may be due to several genetic factors [30] and could justify some of the differences observed between male and female PD patients [30,31,32]. Indeed, experiments performed in animal models (such as on rats and monkeys) and neuroimaging measurement on the human brain seem to show that females have a higher number of DA neurons than males, as well as a greater striatal uptake [25,29,31], which could explain the delayed PD onset in women.

Furthermore, transcriptome analysis has allowed for a better understanding in relation to genes or loci potentially associated with the disease. Several experiments conducted on post-mortem brain tissue of PD patients and related controls, mainly on SN whole tissue or DA neurons obtained with laser capture microdissection (LMD), demonstrated differential gene expression profiles between PD patients and controls [33,34,35,36,37,38,39,40,41,42].

In a previous study, we used Transcriptome Mapper (TRAM) software to analyze a large amount of publicly available microarray data from independent studies conducted on the SN brain region of PD patients and controls, analyzing both post-mortem whole tissue and isolated LMD DA neuron expression data [43]. Thanks to original methods for parsing, normalizing and statistically analyzing expression data, the software was able to generate maps showing differential expression between two sample groups and to identify chromosomal segments and gene clusters which are relevant for different biological conditions [44].

To date only a few gene expression studies have focused on the characterization of transcriptome in male and female PD patients. Such studies, conducted on LMD DA neurons, have highlighted a sex-specific gene expression profile and showed a predominantly male deregulation of the genes involved in the DA neurons degenerative pathways or in mechanisms already associated with the pathogenesis of PD, thus confirming male sex as a risk factor for the disease [41,42].

We believe that further investigations in male and female brain transcriptome profiles could therefore contribute to a better understanding of the physiological and pathological molecular pathways and enable the development of sex-specific therapies.

For this reason, we conducted a new TRAM meta-analysis study to better define the differences in gene expression of PD patients vs. controls, when comparing men and women data sets from SN whole tissue.

## 2. Materials and Methods

### 2.1. Database Search and Selection

We conducted our search for gene expression data related to SN whole tissue of PD patients and healthy controls using the genomics repositories Gene Expression Omnibus (GEO) [45] and ArrayExpress [46]: in GEO with the search terms “Parkinson Disease” and “Homo sapiens”[Organism]; in the ArrayExpress database with the terms “Parkinson Disease” and then filtered for “Homo Sapiens”[Organism], “RNA assay” and “array assay”[Experiment type] and “all array”. We then selected those series including microarray experiments conducted on SN whole tissue and where sex was specified.

A further search was carried out using the criteria described in the TRAM guidelines [44] and listed in our previous work [43]. In particular, all the selected experiments were carried out on specific brain structure and clinical conditions (SN pars compacta from post-mortem brains from individuals with PD and matched controls), based on availability of raw or pre-processed data (see Supplementary Table S1 for details). We did not include data from exon array or other probes, as a too high number of data rows could interfere with program execution. Other exclusion criteria were also: the absence of identifiers corresponding to those found in the GEO sample records (GSM) or Array express sample records; platforms without standard format (for example with an atypical number of genes, i.e., <5000 or >60,000); data with expression values not evidently marked as linear or logarithmic.

Searches were executed up to July 2017.

### 2.2. Literature Search

We performed a systematic literature search to identify further articles related to global gene expression profile experiments in PD patients that were not present in GEO or ArrayExpress. A general search was conducted on PubMed up to July 2017, using the terms “Parkinson disease” and “microarray analysis” and “human”. The medical subject headings (MeSH) terms “Parkinson disease”, “microarray analysis” (or “gene expression profiling” or “oligonucleotide array sequence analysis”), “substantia nigra” and “human” were also used for a more advanced search. The search did not detect additional data.

### 2.3. Tram Analysis

Transcriptome Mapper software is freely available at http://apollo11.isto.unibo.it/software.

We used the TRAM version 1.2 (April 2014) [44] which contains an updated version of UniGene and Entrez Genes databases compared to the original 2011 version [44].

At first, all platforms used in the array experiment that are not present in the TRAM 1.2 version were manually extracted and imported, so that, during the import phase, the software could associate a specific gene symbol via UniGene parsing with each probe identifier.

In order to be imported and analyzed by TRAM, all samples selected for each series were downloaded as tab-delimited text format. Samples were then divided into pools, according to different sex and brain condition: SN from male control subjects (pool A); SN from female control subjects (pool B); SN from male PD-affected patients (pool C); SN from female PD-affected patients (pool D).

As previously stated, when importing the data, the software was able to assign the appropriate gene symbol to each probe identifier through UniGene parsing and subsequently normalize them (via intra- and inter-sample normalization) in order to compare gene expression data from experiments performed with different platforms and/or biological conditions [44]. It is noteworthy that the TRAM software evaluates the possible risk of bias as it is intrinsically resistant to the systematic differences between batches (groups) of samples [44].

Four different analyses were performed according to standard setting (“Map” mode) using default parameters [43,44,47,48,49] and named as below:

- TRAM control (CTR), comparing the transcriptome map of SN from male control subjects (pool A) and SN from female control subjects (pool B);
- TRAM PD, comparing the transcriptome map of SN from male patients affected by PD (pool C) and SN from female patients affected by PD (pool D);
- TRAM MALE, comparing the transcriptome map of SN from male patients affected by PD (pool C) and SN from male control subjects (pool A);
- TRAM FEMALE, comparing the transcriptome map of SN from female patients affected by PD (pool D) and SN from female control subjects (pool B).

Briefly, the “Map” mode analysis was set to evaluate segments of 500,000 bp with a sliding window of 250,000 bp [44]. The expression value for each genomic segment is calculated as the mean of the expression values of all the loci included in that segment. As a result, TRAM highlighted those segments where the expression value was significantly different between the two considered conditions and contained at least three over/under-expressed genes (genes at the top/bottom 2.5% percentile of values) [44].

The statistical significance was calculated taking into account all genes in the genome (genome median) and corrected for multiple comparisons possible causing false discovery rate (FDR) due to the high number of segments or genes in a genome (*q*-value). A segment or a gene was considered to be statistically significantly over- or under-expressed for *q* < 0.05. [44].

### 2.4. Other Analyses

European Bioinformatics Institute (EMBL-EBI) Expression Atlas [50], UniGene [51], National Center for Biotechnology Information (NCBI) Entrez Gene [52] and Gene Ontology (GO) [53] were used to obtain gene-specific information and to functionally characterize the set of genes derived from the TRAM analysis.

## 3. Results

### 3.1. Database and Literature Search

The gene expression data search in GEO and ArrayExpress data repository, followed by the selection according to the criteria described in the Materials and Methods section, allowed us to include a total of 10 series in the meta-analysis. More details about series and samples are listed in Table 1 and Supplementary Table S1.

### 3.2. Male SN Control Samples vs. Female SN Control Samples (TRAM CTR)

TRAM analysis of pool A (73 male SN control samples) vs. pool B (39 female SN control samples) consisted respectively of 1,798,965 and 1,176,191 data points (gene expression value), corresponding to 36,654 mapped loci (Supplementary Table S2).

Results are listed in Table 2 and highlight the differential expression of nine segments: five containing over-expressed genes and four classified as under-expressed.

As expected, the highest expression ratio was observed for a segment on chromosome Y (Yq11.1 region) including the ncRNA *TTTY15* and the known genes *USP9Y* and *DDX3Y*. *USP9Y* is a member of the peptidase C19 family, encoding for a protein that is similar to ubiquitin-specific proteases; *DDX3Y* is a member of the DEAD-box RNA helicase family.

Conversely, the most under-expressed segment is located on chromosome X (Xq13.2) and significance was detected for the ncRNAs *XIST* and *JPX* and for the EST cluster Hs.720466.

Eleven EST clusters (Hs.731466 EST was validated for coding *UBE2QL1* as seen in the UniGene database search) were found as over- or under-expressed, but not associated with known genes.

### 3.3. Male SN PD Patient Samples vs. Female SN PD Patient Samples (TRAM PD)

Transcriptome Mapper analysis of pool C (67 male SN PD patient samples) vs. pool D (48 female SN PD patient samples) consisted respectively of 1,812,722 and 1,148,193 data points (gene expression value), corresponding to 36,654 mapped loci (Supplementary Table S3).

Results are reported in Table 3 and underline the differential expression of nine segments, only one of which is under-expressed.

As per TRAM CTR results, the first over-expressed segment maps on chromosome Y and includes the significant genes *TTTY15*, *USP9Y* and *DDX3Y*.

Only one segment is reported as under-expressed and is located on chromosome 8 (8p23.1), including three genes of the defensins family (*DEFA4*, *DEFA1* and *DEFA3*).

Eleven expressed sequence tags (EST) clusters (Hs.635716 EST was validated for coding *CDR1-AS1* as seen in the UniGene database search) were found as over- or under-expressed, but not associated with known genes.

### 3.4. Male SN PD Patient Samples vs. Male SN Control Samples (TRAM MALE)

Transcriptome Mapper analysis of pool C (67 male SN PD patient samples) vs. pool A (73 male SN control samples) consisted respectively of 1,812,722 and 1,798,965 data points (gene expression value), corresponding to 36,654 mapped loci (Supplementary Table S4).

The results, as reported in Table 4, underline the deregulation of 12 segments, only two of which are under-expressed.

The segment with the highest expression ratio is located on chromosome 1 (1p22.2), showing as statistically significant *GBP1*, a gene of the GBP family of guanylate binding protein, the protein kinase coding gene *PKN2* and the EST cluster Hs.732899 as statistically significant. Another segment with the same expression ratio (1.31) maps on chromosome 15 (15q21.1) and includes the genes *SHC4*, an SHC adaptor protein 4, *EID1*, an EP300 interacting inhibitor of differentiation and the EST cluster Hs.585204.

The lowest expression ratio was reported for the segment on chromosome 19 comprising the genes *POP4*, encoding for a protein subunit of the small nucleolar ribonucleoprotein complexes, *C19orf12*, encoding for a small transmembrane protein and the ncRNA *LOC284395*.

Seventeen EST clusters (Hs.732899 was validated for coding *GBP2* as seen in the UniGene database search, Hs.98445 for coding *RASA2*, Hs.741442 for coding *GS1-124K5.11*) were found as over- or under-expressed, but not associated with known genes.

### 3.5. Female SN PD Patient Samples *vs.* Female SN Control Samples (TRAM FEMALE)

Transcriptome Mapper analysis of pool D (48 female SN PD patients samples) vs. pool B (39 female SN control samples) consisted respectively of 1,148,193 and 1,176,191 data points (gene expression value), corresponding to 36,654 mapped loci (Supplementary Table S5).

Table 5 are listed the statistically significant segments obtained, six of which are over-expressed and only one segment is under-expressed.

The highest expression ratio was observed for a segment on chromosome 8 (8p23.1) that includes three genes belonging to the Defensins protein family (*DEFA4*, *DEFA1*, *DEFA3*) as over-expressed.

The only under-expressed segment is located on chromosome X (Xq27.1), containing the ncRNA *LINC00632*, the gene *CDR1* named as cerebellar degeneration related protein 1, not yet functionally characterized, and the antisense *CDR1-AS1* (corresponding in Table 4 to Hs.635716).

Four EST clusters (Hs.637575 was validated for coding LOC101928809 as seen in the UniGene database search, C8orf46 for coding *VXN* vexin, Hs.660769 for coding *ARID3A*, Hs.635716 for coding *CDR1-AS*) were found as over- or under-expressed, but not associated with known genes.

## 4. Discussion

The main aim of this study was to characterize the male vs. female gene expression differences observed in the SN brain region of PD patients and matched controls.

Our meta-analysis was conducted on SN whole tissue to assess the global alteration occurring in this brain region, considering the contribution of both DA neurons, whose degeneration leads to the onset of the major (motor and non-motor) symptoms, and glial cells (non-neuronal cells). The TRAM software was chosen for its particular features, mainly for its ability to process microarray data obtained from independent studies performed with different array platforms and for the intra- and inter-sample normalization (scaled quantile) method, useful to avoid possible batch effect [44]. In addition, the statistical validity of the software has been confirmed in different studies [43,47,48,49].

In our previous study, we used TRAM to focus on segments differentially expressed by the disease but not by sex [43]. Starting from the same data collection, plus new microarray series, we specifically investigated the sex differences. In particular, we performed a comparison of healthy controls in order to generate a sex-specific transcriptome reference map of normal substantia nigra (TRAM CTR) to compare with the others of unhealthy tissues (TRAM PD, TRAM MALE and TRAM FEMALE) and possibly evaluate the differences due only to the disease and not to the sex.

### 4.1. Sex-Biased Gene Expression in Parkinson’s and Control SN

The transcriptome map of healthy controls showed the evident different expression in male vs. female SN of segments located on sex chromosomes (TRAM CTR, Table 2). This confirm the robustness of TRAM statistics, as it identifies sample-specific segments. In particular, we can note that the Y chromosome segment, containing the male sex specific genes *TTTY15*, *USP9Y* and *DDX3Y*, is the most over-expressed in both analyses involving the comparison between male vs. female subjects (TRAM CTR and TRAM PD, respectively Table 2 and Table 3).

Therefore, the present study supports the known differential expression of X- and Y-linked genes in a sex-specific brain gene expression analysis [41,55] as well as the obvious deregulation of loci not mapping on sex chromosomes [41,42]. Among them, we can highlight several genes which encode for proteins with specific neuronal function, found to be upregulated in males. i.e., the *MSRA* gene in SN control samples (TRAM CTR, Table 2), whose product has a key role in oxidative stress response and a high expression in the brain, including the SN, with a higher immuno-reactivity in neurons than in glial cells [56,57]; the *SCN3A* and *SCN2A* genes, that encode for glycoprotein members of a family of voltage-gated sodium channels involved in the generation and propagation of action potentials in neurons and muscle cells [58,59]; the *CSRNP3* gene, encoding for a transcriptional activator, whose mouse ortholog Mbu-1 shows a high expression in the central nervous system, in particular in the brain and spinal cord in the course of embryonic development but also in adults [60]; the *NSUN2* gene, whose product is a methyltransferase involved in the stabilization of the anticodon-codon pairing and the correct translation of the mRNA, and whose loss-of-function is associated with increased cellular stress and neurodevelopmental disorders [61].

It is noteworthy that these genes have not yet been reported in relevant research literature as being associated with sex-biased gene expression, so they could be further investigated to better understand the anatomical and functional dimorphism of SN tissue.

Nonetheless, the under-expressed segments in Table 2 reflect the main deregulation of ncRNA genes or unknown transcripts in men, indicating a possible higher activity of epigenetic mechanisms in women. Other studies have pointed out the significance of epigenetic mechanisms in promoting sexual dimorphism in the brain and also their involvement in several neurological and psychiatric disorders, suggesting that epigenetics could have a role in the different regulation and susceptibility of these diseases [62].

In the TRAM PD analysis (Table 3), in addition to the confirmed presence of the segment on chromosome Y, we can also note the over-expression of *GABRB2*, *GABRA6*, and *GABRA1* genes, encoding for various subunits of a chlorine channel that mediates the rapid inhibition of synaptic transmission in the central nervous system. Gamma-aminobutyric acid GABA is the major inhibitory neurotransmitter of the brain. Sex differences in brain neurochemistry have been well documented, with studies reporting GABAergic variations together with DA and serotonergic systems alterations [31]. In addition, in vitro and in vivo studies are focused on the relationship between deregulation of GABA receptors and PD [63,64]. In particular, it has been assumed that a decrease in GABA receptors in the dentate nucleus is related to tremors generation [65]. As shown by the results of the TRAM PD analysis, the under-expression of some GABA receptors genes in females could suggest that the early tremor symptom in women could be associated with the down-regulation of the GABA system.

Furthermore, in Table 3 we connote another nervous tissue-associated gene, *CDR1* (cerebellar degeneration related protein 1) and the antisense *CDR1-AS1* (Hs.635716), both of which are over-expressed in male PD. These two loci are also reported as significant in the TRAM FEMALE analysis (Table 5), where they are under-expressed in female PD vs. control subjects. Recent studies described *CDR1* and its antisense counterpart as highly expressed in the brain [66]. *CDR1-AS1* is a circular RNA (circRNA) that binds and inhibits microRNA (miRNA) such as miR-7, which are in turn involved in alpha synuclein repression; in particular, this mechanism has been investigated in humans and mice, where the loss-of-function of *CDR1-AS1* is associated with brain dysfunctions [67].

If we consider the two specific analyses for PD, TRAM MALE and TRAM FEMALE, the difference between their relative results is noticeable.

In the TRAM FEMALE analysis (Table 5), the main over-expression in pathological tissue of segments including immune system activation-related loci is very clear: i.e., *DEFA4*, *DEFA1* and *DEFA3* genes belonging to the defensins family of proteins and involved in host defense; *CXCL9*, *CXCL10* and *CXCL11* genes, that encode for the Chemokine protein family; *AZU1*, *PRTN3* and *ELANE* genes, known to be expressed coordinately and to encode for proteins incorporated into azurophil granules during neutrophil differentiation.

When we compare all the affected males vs. females in the TRAM PD analysis, the only under-expressed segment is the one on chromosome 8 including the defensins gene family, confirming the sexually dimorphic activation of gliosis/inflammation specific genes in PD female subjects. Some of these genes were already commented on in our previous work [43] and several studies described the activation of microglia and increased expression of immune system mediators in the brain as a common feature of many neurodegenerative diseases, including PD [68,69,70]. Notably, recent findings have associated higher brain levels of inflammatory markers with more severe symptoms of stress-related depression and fatigue [71] and suggested that sex differences in neuroinflammation may explain the female bias in these symptoms [72]. Based on these data, we can hypothesize that depression and anxiety which mainly characterize females affected by PD could be related to the alteration of inflammatory pathways.

In the TRAM MALE analysis, the results highlight several genes that cannot be related to common functional pathways. Among them, we can underline as over-expressed the *GPNMB* gene (glycoprotein nmb) whose locus has been indicated as a susceptibility factor for PD in gene expression and methylation assays [73]. The locus is also present in the “Top Results” list of PDGene, a publicly available database providing collection of genome wide association studies (GWAS) performed on PD phenotypes [74]. Interestingly, other segments resulted in our study seem to include genes with genetic variant associated to the disease. Further investigation integrating GWAS and gene expression data, could be a robust approach for the identification of candidate genes and functions associated.

### 4.2. Other Results

It is interesting to observe the high number of Hs. sequences shown to be under- or over-expressed in a statistical manner. These sequences are from H. sapiens transcriptome, still not fully characterized and supposedly coding for proteins or non-coding transcripts, such as lncRNA, miRNA or other small non-coding RNAs (sncRNAs). Also noticeable is the presence of LOC sequences (genes of uncertain function) and large intergenic non-coding RNAs (lincRNAs), recently emerging as key regulators of several cellular processes. Some evidence suggests their possible function in the central nervous system as regulators in brain evolution and neuronal development [75]. It is worthwhile considering that the *Homo sapiens* (Hs.). sequences are constantly updated in the UniGene database, and they could be linked to gene sequences in the future.

One of the TRAM software’s main features is to detect the differential expression of a chromosomal segment region, not just single genes, providing statistical analysis of over- or under-expressed regions when compared to the whole genome or to the relative chromosome [44]. Results could suggest common regulatory mechanisms for the many genes within the significant segment, whose genetic variability has already been associated with PD.

Remarkably, the present study identifies some segments immediately adjacent to known PD-related genes, i.e., the segment on chromosome 4 (4q21.1) that in the TRAM FEMALE analysis (Table 5), was shown to be adjacent to the one comprising the *SNCA* gene (*PARK1*/*PARK4*), encoding for alpha-synuclein. Currently, this protein is one of the main pathophysiological hallmarks of PD since its accumulation forms the typical intracellular aggregates characteristic of some neurodegenerative diseases, the LBs [8,9,10,11,12].

In the same analysis, we can note the segment on chromosome 1 (1q21) known to be the location of the *GBA* gene (glucosylceramidase β), another important risk factor for PD and dementia with LBs [12,76]. In addition, in the TRAM CTR analysis the segment on chromosome 2 was over-expressed in men vs. women (2q24) and including the *SCN3A* gene as statistically significant, it is very near to *SKT39* (serine/threonine kinase 39), a gene recently confirmed as associated with PD in a genome-wide association study [77]. Interestingly, the same study also indicates also *SCN3A* as a novel risk locus.

## 5. Conclusions

Taken together, the TRAM meta-analysis results lead us to hypothesize a possible activation of specific sex-biased systems in PD susceptibility, although further investigations are needed to confirm the hypothesis.

Most notably, these data could suggest the existence of differences in the activation of neurodegeneration and neuroinflammatory mechanisms. In particular, the under-expression of some GABA receptors genes observed in females could be related to the prevalence of tremors as their initial symptom. In addition, the increased expression of proinflammatory genes detected in women could explain the different development of the PD in the two sexes, with females shown to be more likely to be affected by depression and anxiety, symptoms that reduce patients’ quality of life.

The validation of the molecular pathways and genes which proved to be significant in this study could help to understand the etiology of PD and contribute in identifying those factors underpinning differences between men and women. Having a better understanding of the genes involved in the disease could also be helpful in the design of new drugs to slow disease progression and to potentially define sex-specific therapies.

Finally, the TRAM software has provided some confirmation and some new insights into PD, proving to be a useful tool in the study of a complex disease, where the need to integrate different types of genetic technologies and approaches is fully acknowledged.

## Figures and Tables

**Table 1:** Main features of the samples selected for the meta-analysis with Transcriptome Mapper (TRAM) software.

GEO Series	GEO Platform ID	CTR/PD	Sex	Samples	Age at Death (Range)	Pool	PMID
GSE26927	GPL6255	CTR	M	6/118	58.7 (54–66)	A	22864814 [33]
		CTR	F	2/118	82 (60–104)	B	
		PD	M	6/118	82.5 (76–87)	C	
		PD	F	6/118	80.6 (77–86)	D	
GSE20333	GPL201	CTR	M	5/12	77.6 (68–88)	A	15455214 [34]
		CTR	F	1/12	86	B	
		PD	M	2/12	72.5 (70–75)	C	
		PD	F	4/12	79.3 (73–87)	D	
GSE8397	GPL96	CTR	M	10/48	65.1 (46–77)	A	16344956 [35]
		CTR	F	4/48	78.5 (77–80)	B	
		PD	M	10/48	77.4 (68–83)	C	
		PD	F	8/48	84.7 (81–87)	D	
GSE8397	GPL97	CTR	M	10/48	65.1 (46–77)	A	16344956 [35]
		CTR	F	4/48	78.5 (77–80)	B	
		PD	M	10/48	77.4 (68–83)	C	
		PD	F	8/48	84.7 (81–87)	D	
GSE7621	GPL570	CTR	M	4/25	-	A	17571925 [36]
		CTR	F	5/25	-	B	
		PD	M	13/25	-	C	
		PD	F	3/25	-	D	
GSE20292	GPL96	CTR	M	13/29	63 (41–82)	A	15965975 [37]
		CTR	F	5/29	76.8 (72–94)	B	
		PD	M	6/29	72.5 (67–80)	C	
		PD	F	5/29	79 (70–84)	D	
GSE20164	GPL96	CTR	M	1/11	90	A	20926834 [38]
		CTR	F	4/11	78 (72–88)	B	
		PD	M	4/11	82(74–87)	C	
		PD	F	2/11	80.5 (79–82)	D	
GSE20163	GPL96	CTR	M	7/17	69 (52–84)	A	20926834 [38]
		CTR	F	1/17	71	B	
		PD	M	3/17	81.3 (80–84)	C	
		PD	F	1/17	70	D	
GSE20159	GPL6497	CTR	M	9/33	74.8 (40–95)	A	20926834 [38]
		CTR	F	8/33	74.5 (45–87)	B	
		PD	M	8/33	77.1 (56–94)	C	
		PD	F	8/33	78.2 (78–103)	D	
GSE3526	GPL570	CTR	M	4/353	32 (25–35)	A	16572319 [39]
		CTR	F	4/353	40.7 (23–53)	B	
GSE43490	GPL6480	CTR	M	4/41	69.2 (58–85)	A	25525598 [40]
		CTR	F	1/41	70	B	
		PD	M	5/41	74 (64–90)	C	
		PD	F	3/41	83 (80–85)	D	

Samples involved in the sex-specific meta-analysis of Parkinson's disease (PD) gene expression data. Gene Expression Omnibus (GEO) series and platform ID: ID numbers as stated in the GEO database; CTR/PD: Control/PD patient sample; Sex: Male (M) and female (F) ; Samples: Number of samples selected for each series; Age at death: Mean value of all the indicated patient/control age at death (the age range was also reported); Pool: In order to perform the described analysis all samples were divided into pools, based on patients’ sex and brain condition (see Materials and Methods Section, TRAM analysis section); PMID: PubMed identifier number of the reference reported in the GEO database. Additional details about series and samples are listed in Supplementary Table S1.

**Table 2:** Chromosomal segments statistically significant in TRAM “Map” mode analysis of male substantia nigra (SN) control samples vs. female SN control samples (TRAM CTR).

Chr	Location	Segment Start	Segment End	Expression Ratio	*q*-Value	Genes in the Segment
ChrY	Yq11.1 *	12,500,001	13,000,000	2.09	1.20E-04	Hs.161318*+ **TTTY15+ USP9Y+ DDX3Y+***
Chr8	8p23.1	9,750,001	10,250,000	1.57	2.20E-04	*TNKS+ **LINC00599+*****Hs.566938+ *MSRA+*** Hs.669947−
Chr2	2q24 *	165,000,001	165,500,000	1.48	1.00E-05	***Hs.541684+ SCN3A+ SCN2A+*** *Hs.541682+ **CSRNP3+***
Chr5	5p15.31 *	6,250,001	6,750,000	1.37	7.00E-05	Hs.582434+ Hs.544271− FLJ33360− Hs.360957− ***MED10+*** Hs.561435+ ***UBE2QL1+*** **Hs.731466+** Hs.734710− ***NSUN2+SRD5A1+** LOC100505625− PAPD7+* Hs.669866+ Hs.656059−
Chr1	1p22.2 *	90,250,001	90,750,000	0.77	6.70E-04	Hs.253993− **Hs.571857−** Hs.441432− Hs.308351+ **Hs.212483−** *BARHL2−* **Hs.576073−**
Chr6	6q14.1 *	75,500,001	76,000,000	0.74	6.70E-04	Hs.583003− *SENP6−* **Hs.597951−** Hs.661961− **Hs.666419−** ***MYO6−*** *IMPG1−*
Chr2	2q33.3	205,500,001	206,000,000	0.73	6.70E-04	*PARD3B−***Hs.271903−***NRP2−* Hs.663547− *INO80D+* **Hs.713983− Hs.610328−**
ChrX	Xq13.2	73,750,001	74,250,000	0.70	2.89E-03	***XIST−JPX−*** Hs.611693− **Hs.720466−** Hs.648496− *FTX−* Hs.619548+ *BMP2KL−* Hs.276976− Hs.607917− Hs.742329− Hs.729494−

Statistically significant segments obtained with TRAM “Map” mode analysis (default parameters) comparing pool A (SN samples from male controls) vs. pool B (SN samples from female controls). Segments are sorted by decreasing the Expression Ratio. Chr: Chromosome; Location: Segment cytoband derived from the first mapped gene within the segment; Segment Start/End: Chromosomal coordinates for each segment; Genes in the segment: Bold and +: Over-expressed gene; Bold and −: Under-expressed gene; ‘+’ or ‘−’: Gene expression value higher or lower than the median value, respectively. UniGene EST clusters (named with the prefix Hs. for *Homo sapiens*) with an expression value are also reported in Table 2. * Cytoband was derived from the UCSC Genome Browser [54].

**Table 3:** Chromosomal segments statistically significant in TRAM “Map” mode analysis of male SN PD patient sample vs. female SN PD patient samples (TRAM PD).

Chr	Location	Segment Start	Segment End	Expression Ratio	*q*-Value	Genes in the Segment
ChrY	Yq11.1 *	12,500,001	13,000,000	2.97	1.22E-04	Hs.161318+ ***TTTY15+ USP9Y+ DDX3Y+***
Chr5	5q34	161,500,001	162,000,000	1.97	1.24E-04	***GABRB2+ GABRA6+ GABRA1+***
Chr2	2p22.3 *	34,500,001	35,000,000	1.58	1.22E-04	**Hs.580379+ Hs.551445+** Hs.542103+ **Hs.542102+**
ChrX	Xq27.1	140,500,001	141,000,000	1.49	2.00E-04	***SOX3+****LINC00632+***Hs.635716+*CDR1+*** Hs.562002+
Chr3	3p21 *	41,000,001	41,500,000	1.40	1.77E-04	**Hs.147846+** Hs.581542− **Hs.565967**+ Hs.581431+ ***CTNNB1+*** Hs.658202+ **Hs.622506+** *ULK4−* Hs.543182+
Chr16	16p13.3	6,750,001	7,250,000	1.40	3.36E-04	***RBFOX1+*** Hs.737046− **Hs.606954+** Hs.675515− Hs.680357+ **Hs.655766+**
Chr3	3q26.1	167,500,001	168,000,000	1.33	7.93E-04	*WDR49−* Hs.572920+ **Hs.171377+** Hs.589980+ *PDCD10+ SERPINI1+* **Hs.575028+ Hs.542878+**
Chr8	8p23.1	6,750,001	7,250,000	0.77	3.69E-03	*AGPAT5+* Hs.545543− *XKR5+ DEFB1− DEFA6−* Hs.583889− ***DEFA4− DEFA1− DEFA3−** DEFA5−* Hs.583868− Hs.552561− Hs.545542+

Statistically significant segments obtained with TRAM “Map” mode analysis (default parameters) comparing pool C (SN samples from males affected by PD) vs. pool D (SN samples from females affected by PD). Segments are sorted by decreasing the Expression Ratio. Chr: Chromosome; Location: Segment cytoband derived from the first mapped gene within the segment; Segment Start/End: Chromosomal coordinates for each segment; Genes in the segment: Bold and +: Over-expressed gene; Bold and −: Under-expressed gene; ‘+’ or ‘−’: Gene expression value higher or lower than the median value, respectively. UniGene EST clusters (named with the prefix Hs. for *Homo sapiens*) with an expression value are also reported in Table 3. * Cytoband was derived from the UCSC Genome Browser [54].

**Table 4:** Chromosomal segments statistically significant in TRAM “Map” mode analysis of male SN PD patient samples vs. male SN control samples (TRAM MALE).

Chr	Location	Segment Start	Segment End	Expression Ratio	*q*-Value	Genes in the Segment
Chr1	1p22.2	88,750,001	89,250,000	1.31	4.02E-03	***PKN2+*** Hs.537994+ *GTF2B− CCBL2+* Hs.562190+ *GBP3+ **GBP1+*** Hs.205458− Hs.170957− **Hs.732899+** *GBP2+ GBP7− GBP4+*
Chr15	15q21.1	48,500,001	49,000,000	1.31	1.36E-03	*FBN1+ Hs.610605+ CEP152+* **Hs.585204+ *SHC4+ EID1+*** *Hs.714048+ SECISBP2L+*
Chr5	5q15	95,750,001	96,250,000	1.27	1.56E-03	***RHOBTB3+****GLRX+***Hs.543999−** Hs.543998− *C5orf27+ ELL2+* **Hs.608694+** **Hs.656129+** Hs.259567+
Chr13	13q32.1	97,000,001	97,500,000	1.26	6.50E-04	***MBNL2+*Hs.660409+ Hs.662697+** Hs.672758+ Hs.657782+ **Hs.732232+** Hs.577859− Hs.326718+ Hs.539599+ Hs.539598+ *RAP2A+* Hs.646603+
Chr18	18q21.33	62,500,001	63,000,000	1.26	9.00E-04	ZCCHC2+ Hs.656351+ Hs.333785− ***PHLPP1+*** **Hs.679153+**
Chr3	3q26.1	167,500,001	168,000,000	1.26	1.36E-03	*WDR49−* Hs.572920+ **Hs.171377+ Hs.589980+** *PDCD10+ SERPINI1−* Hs.575028+ **Hs.542878+**
Chr3	3q22-q23	141,500,001	142,000,000	1.23	5.30E-04	***RASA2+* Hs.98445+ Hs.603855+** *LOC646730+ RNF7− GRK7− ATP1B3+* **Hs.660667+** *TFDP2+*
Chr7	7p15	23,250,001	23,750,000	1.23	6.90E-04	***GPNMB+****MALSU1+ IGF2BP3+* Hs.29733− *TRA2A+* **Hs.644466+** **Hs.608901+** Hs.743502+ *CCDC126+* Hs.737536+ Hs.128757+ ***FAM221A+** STK31+*
Chr12	12q15	70,250,001	70,750,000	1.23	1.21E-03	*CNOT2+***Hs.663352+** Hs.577543− ***KCNMB4+*** **Hs.665051+** *PTPRB+ PTPRR−*
Chr7	7q11.21	66,000,001	66,500,000	0.69	3.00E-05	***ASL−*** *CRCP+ **TPST1−** Hs.677061− **LINC00174−*** **Hs.741442−** *LOC100289098+*
Chr19	19q12	29,250,001	29,750,000	0.61	1.50E-04	***LOC284395−******POP4−** PLEKHF1+ **C19orf12−*** Hs.551392−

Statistically significant segments obtained with TRAM “Map” mode analysis (default parameters) comparing pool C (SN samples from males affected by PD) vs. pool A (SN samples from male control subjects). Segments are sorted by decreasing the Expression Ratio. Chr: Chromosome; Location: Segment cytoband derived from the first mapped gene within the segment; Segment Start/End: Chromosomal coordinates for each segment; Genes in the segment: Bold and +: Over-expressed gene; Bold and −: Under-expressed gene; ‘+’ or ‘−’: Gene expression value higher or lower than the median value respectively. UniGene EST clusters (named with the prefix Hs. for *Homo sapiens*) with an expression value are also reported in Table 4.

**Table 5:** Chromosomal segments statistically significant in TRAM “Map” mode analysis of female SN PD patient sample vs. female SN control samples (TRAM FEMALE).

Chr	Location	Segment Start	Segment End	Expression Ratio	*q*-Value	Genes in the Segment
Chr8	8p23.1	6,750,001	7,250,000	1.38	5.53E-03	*AGPAT5−* Hs.545543+ *XKR5− DEFB1+ DEFA6−* Hs.583889+***DEFA4+ DEFA1+ DEFA3+** DEFA5−* Hs.583868+ Hs.552561+ Hs.545542−
Chr4	4q21.1	75,750,001	76,250,000	1.17	5.92E-03	*USO1+* Hs.662684+ *PPEF2+ NAAA+* Hs.633144+ *SDAD1+* **Hs.637575− *CXCL9+*** *ART3+ **CXCL10+CXCL11+** NUP54+ SCARB2+ FAM47E−*
Chr2	2q33.1	197,250,001	197,750,000	1.16	1.43E-03	*ANKRD44+* Hs.687027+ Hs.570284+ **Hs.471011+** *SF3B1− COQ10B+ HSPD1+ **HSPE1+** MOB4−* **Hs.595504+** ***RFTN2+*** Hs.676585− *MARS2+ BOLL−*
Chr1	1q21.1	149,500,001	150,000,000	1.16	2.01E-03	*LINC00869+ HIST2H2BF− FCGR1A−* Hs.624313+ ***HIST2H3D+*** Hs.741509− Hs.528615− *HIST2H4A+* **Hs.597557+** *HIST2H3C− HIST2H2AA3+* **Hs.713638+** *HIST2H2AA4− HIST2H4B+ **HIST2H2BE+** HIST2H2AC+ HIST2H2AB− BOLA1− SV2A− SF3B4+ MTMR11+* **Hs.194081+** *OTUD7B+*
Chr8	8q13.1	66,500,001	67,000,000	1.13	4.97E-03	***C8orf46+****MYBL1+* Hs.732745+ *VCPIP1+ C8orf44+* Hs.734405− *SGK3+ **MCMDC2+SNHG6+** TCF24+ PPP1R42+*
Chr19	19p13.3	500,001	1,000,000	1.12	3.51E-02	*MADCAM1− TPGS1− CDC34+ GZMM−* Hs.214491− *BSG+ HCN2− FLJ45684+ POLRMT+ FGF22− RNF126+* **Hs.638433−** *FSTL3− PRSS57+ PALM+ MISP− PTBP1+ LPPR3− **AZU1+ PRTN3+ELANE+** CFD+ MED16+ R3HDM4+ KISS1R− ARID3A+* Hs.735832+ **Hs.660769−** *WDR18+*
ChrX	Xq27.1	140,500,001	141,000,000	0.65	1.50E-04	*SOX3− **LINC00632−*****Hs.635716−*CDR1−*** Hs.562002+

Statistically significant segments obtained with TRAM “Map” mode analysis (default parameters) comparing pool D (SN samples from females affected by PD) vs. pool B (SN samples from female control subjects). Segments are sorted by decreasing the Expression Ratio. Chr: Chromosome; Location: Segment cytoband derived from that of the first mapped gene within the segment; Segment Start/End: Chromosomal coordinates for each segment; Genes in the segment: Bold and +: Over-expressed gene; Bold and −: Under-expressed gene; ‘+’ or ‘−’: Gene expression value higher or lower than the median value, respectively. UniGene EST clusters (named with the prefix Hs. for *Homo sapiens*) with an expression value are also reported in Table 5.

## Supplementary Materials

The following are available online at http://www.mdpi.com/2073-4425/9/6/275/s1, Table S1: Samples selected for the meta-analysis of gene expression profiles of PD patients vs. healthy controls, Table S2: List of 36,654 TRAM mapped loci for which an expression value A/B was calculated (SN samples from male controls vs. female controls), Table S3: List of 36,654 TRAM mapped loci for which an expression value C/D was calculated (SN samples from PD male patients vs. PD female patients), Table S4: List of 36,654 TRAM mapped loci for which an expression value C/A was calculated (SN samples from PD male patients vs. male controls), Table S5: List of 36,654 TRAM mapped loci for which an expression value D/B was calculated (SN samples from PD female patients vs. female controls).

## References

[1] De Lau L.M., Breteler M.M. (2006). Epidemiology of Parkinson’s disease. Lancet Neurol..

[2] Wirdefeldt K., Adami H.O., Cole P., Trichopoulos D., Mandel J. (2011). Epidemiology and etiology of Parkinson’s disease: A review of the evidence. Eur. J. Epidemiol..

[3] Jankovic J. (2008). Parkinson’s disease: Clinical features and diagnosis. J. Neurol. Neurosurg. Psychiatry.

[4] Rodriguez-Oroz M.C., Jahanshahi M., Krack P., Litvan I., Macias R., Bezard E., Obeso J.A. (2009). Initial clinical manifestations of Parkinson’s disease: Features and pathophysiological mechanisms. Lancet Neurol..

[5] Vernier P., Moret F., Callier S., Snapyan M., Wersinger C., Sidhu A. (2004). The degeneration of dopamine neurons in Parkinson’s disease: Insights from embryology and evolution of the mesostriatocortical system. Ann. N. Y. Acad. Sci..

[6] Lesage S., Brice A. (2009). Parkinson’s disease: From monogenic forms to genetic susceptibility factors. Hum. Mol. Genet..

[7] Gibb W.R., Lees A.J. (1988). The relevance of the Lewy body to the pathogenesis of idiopathic Parkinson’s disease. J. Neurol. Neurosurg. Psychiatry.

[8] Hernandez D.G., Reed X., Singleton A.B. (2016). Genetics in Parkinson disease: Mendelian versus non-Mendelian inheritance. J. Neurochem..

[9] Mullin S., Schapira A. (2015). The genetics of Parkinson’s disease. Br. Med. Bull..

[10] Dexter D.T., Jenner P. (2013). Parkinson disease: From pathology to molecular disease mechanisms. Free Radic. Biol. Med..

[11] Coppedè F. (2012). Genetics and epigenetics of Parkinson’s disease. Sci. World J..

[12] Schulte C., Gasser T. (2011). Genetic basis of Parkinson’s disease: Inheritance, penetrance, and expression. Appl. Clin. Genet..

[13] Lardenoije R., Iatrou A., Kenis G., Kompotis K., Steinbusch H.W., Mastroeni D., Coleman P., Lemere C.A., Hof P.R., van den Hove D.L. (2015). The epigenetics of aging and neurodegeneration. Prog. Neurobiol..

[14] Marques S.C., Oliveira C.R., Pereira C.M., Outeiro T.F. (2011). Epigenetics in neurodegeneration: A new layer of complexity. Prog. Neuropsychopharmacol. Biol. Psychiatry.

[15] Kontopoulos E., Parvin J.D., Feany M.B. (2006). α-Synuclein acts in the nucleus to inhibit histone acetylation and promote neurotoxicity. Hum. Mol. Genet..

[16] Matsumoto L., Takuma H., Tamaoka A., Kurisaki H., Date H., Tsuji S., Iwata A. (2010). CpG demethylation enhances α-synuclein expression and affects the pathogenesis of Parkinson’s disease. PLoS ONE.

[17] Harrison I.F., Dexter D.T. (2013). Epigenetic targeting of hystone deacetylase: Therapeutic potential in Parkinson’s disease?. Pharmacol. Ther..

[18] Guhathakurta S., Bok E., Evangelista B.A., Kim Y.S. (2017). Deregulation of α-synuclein in Parkinson’s disease: Insight from epigenetic structure and transcriptional regulation of SNCA. Prog. Neurobiol..

[19] Walden H., Muqit M.M. (2017). Ubiquitin and Parkinson’s disease through the looking glass of genetics. Biochem. J..

[20] Majidinia M., Mihanfar A., Rahbarghazi R., Nourazarian A., Bagca B., Avci Ç.B. (2016). The roles of non-coding RNAs in Parkinson’s disease. Mol. Biol. Rep..

[21] Rodriguez M., Rodriguez-Sabate C., Morales I., Sanchez A., Sabate M. (2015). Parkinson’s disease as a result of aging. Aging Cell.

[22] Gillies G.E., Pienaar I.S., Vohra S., Qamhawi Z. (2014). Sex differences in Parkinson’s disease. Front. Neuroendocrinol..

[23] Smith K.M., Dahodwala N. (2014). Sex differences in Parkinson’s disease and other movement disorders. Exp. Neurol..

[24] Loke H., Harley V., Lee J. (2015). Biological factors underlying sex differences in neurological disorders. Int. J. Biochem. Cell Biol..

[25] Wooten G.F., Currie L.J., Bovbjerg V.E., Lee J.K., Patrie J. (2004). Are men at greater risk for Parkinson’s disease than women?. J. Neurol. Neurosurg. Psychiatry.

[26] Taylor K.S., Cook J.A., Counsell C.E. (2007). Heterogeneity in male to female risk for Parkinson’s disease. J. Neurol. Neurosurg. Psychiatry.

[27] Miller I.N., Cronin-Golomb A. (2010). Gender differences in Parkinson’s disease: Clinical characteristics and cognition. Mov. Disord..

[28] Saunders-Pullman R. (2003). Estrogens and Parkinson disease: Neuroprotective, symptomatic, neither, or both?. Endocrine.

[29] Küppers E., Ivanova T., Karolczak M., Beyer C. (2000). Estrogen: A multifunctional messenger to nigrostriatal dopaminergic neurons. J. Neurocytol..

[30] Panzica G., Melcangi R.C. (2016). Structural and molecular brain sexual differences: A tool to understand sex differences in health and disease. Neurosci. Biobehav. Rev..

[31] Cosgrove K.P., Mazure C.M., Staley J.K. (2007). Evolving knowledge of sex differences in brain structure, function, and chemistry. Biol. Psychiatry.

[32] Sun Y., Lee R., Chen Y., Collinson S., Thakor N., Bezerianos A., Sim K. (2015). Progressive gender differences of structural brain networks in healthy adults: A longitudinal, diffusion tensor imaging study. PLoS ONE.

[33] Durrenberger P.F., Fernando F.S., Magliozzi R., Kashefi S.N., Bonnert T.P., Ferrer I., Seilhean D., Nait-Oumesmar B., Schmitt A., Gebicke-Haerter P.J. (2012). Selection of novel reference genes for use in the human central nervous system: A BrainNet Europe Study. Acta Neuropathol..

[34] Grünblatt E., Mandel S., Jacob-Hirsch J., Zeligson S., Amariglo N., Rechavi G., Li J., Ravid R., Roggendorf W., Riederer P. (2004). Gene expression profiling of parkinsonian substantia nigra pars compacta; alterations in ubiquitin-proteasome, heat shock protein, iron and oxidative stress regulated proteins, cell adhesion/cellular matrix and vesicle trafficking genes. J. Neural Transm..

[35] Moran L.B., Duke D.C., Deprez M., Dexter D.T., Pearce R.K., Graeber M.B. (2006). Whole genome expression profiling of the medial and lateral substantia nigra in Parkinson’s disease. Neurogenetics.

[36] Lesnick T.G., Papapetropoulos S., Mash D.C., Ffrench-Mullen J., Shehadeh L., de Andrade M., Henley J.R., Rocca W.A., Ahlskog J.E., Maraganore D.M. (2007). A genomic pathway approach to a complex disease: Axon guidance and Parkinson disease. PLoS Genet..

[37] Zhang Y., James M., Middleton F.A., Davis R.L. (2005). Transcriptional analysis of multiple brain regions in Parkinson’s disease supports the involvement of specific protein processing, energy metabolism, and signaling pathways, and suggests novel disease mechanisms. Am. J. Med. Genet. B Neuropsychiatr. Genet..

[38] Zheng B., Liao Z., Locascio J.J., Lesniak K.A., Roderick S.S., Watt M.L., Eklund A.C., Zhang-James Y., Kim P.D., Hauser M.A. (2010). PGC-1α, a potential therapeutic target for early intervention in Parkinson’s disease. Sci. Transl. Med..

[39] Roth R.B., Hevezi P., Lee J., Willhite D., Lechner S.M., Foster A.C., Zlotnik A. (2006). Gene expression analyses reveal molecular relationships among 20 regions of the human CNS. Neurogenetics.

[40] Corradini B.R., Iamashita P., Tampellini E., Farfel J.M., Grinberg L.T., Moreira-Filho C.A. (2014). Complex network-driven view of genomic mechanisms underlying Parkinson’s disease: Analyses in dorsal motor vagal nucleus, locus coeruleus, and substantia nigra. Biomed. Res. Int..

[41] Cantuti-Castelvetri I., Keller-McGandy C., Bouzou B., Asteris G., Clark T.W., Frosch M.P., Standaert D.G. (2007). Effects of gender on nigral gene expression and Parkinson disease. Neurobiol. Dis..

[42] Simunovic F., Yi M., Wang Y., Stephens R., Sonntag K.C. (2010). Evidence for gender-specific transcriptional profiles of nigral dopamine neurons in Parkinson disease. PLoS ONE.

[43] Mariani E., Frabetti F., Tarozzi A., Pelleri M.C., Pizzetti F., Casadei R. (2016). Meta-analysis of Parkinson’s disease transcriptome data using TRAM software: Whole substantia nigra tissue and single dopamine neuron differential gene expression. PLoS ONE.

[44] Lenzi L., Facchin F., Piva F., Giulietti M., Pelleri M.C., Frabetti F., Vitale L., Casadei R., Canaider S., Bortoluzzi S. (2011). TRAM (Transcriptome Mapper): Database-driven creation and analysis of transcriptome maps from multiple sources. BMC Genom..

[45] Barrett T., Wilhite S.E., Ledoux P., Evangelista C., Kim I.F., Tomashevsky M., Marshall K.A., Phillippy K.H., Sherman P.M., Holko M. (2013). NCBI GEO: Archive for functional genomics data sets—Update. Nucleic Acids Res..

[46] Kolesnikov N., Hastings E., Keays M., Melnichuk O., Tang Y.A., Williams E., Dylag M., Kurbatova N., Brandizi M., Burdett T. (2015). ArrayExpress update simplifying data submissions. Nucleic Acids Res..

[47] Caracausi M., Vitale L., Pelleri M.C., Piovesan A., Bruno S., Strippoli P. (2014). A quantitative transcriptome reference map of the normal human brain. Neurogenetics.

[48] Caracausi M., Rigon V., Piovesan A., Strippoli P., Vitale L., Pelleri M.C. (2015). A quantitative transcriptome reference map of the normal human hippocampus. Hippocampus.

[49] Pelleri M.C., Piovesan A., Caracausi M., Berardi A.C., Vitale L., Strippoli P. (2014). Integrated differential transcriptome maps of Acute Megakaryoblastic Leukemia (AMKL) in children with or without Down Syndrome (DS). BMC Med. Genom..

[50] Petryszak R., Keays M., Tang Y.A., Fonseca N.A., Barrera E., Burdett T., Füllgrabe A., Fuentes A.M., Jupp S., Koskinen S. (2016). Expression Atlas update-an integrated database of gene and protein expression in humans, animals and plants. Nucleic Acids Res..

[51] Pontius J.U., Wagner L., Schuler G.D., McEntyre J., Ostell J. (2003). UniGene: A unified view of the transcriptome. The NCBI Handbook.

[52] Brown G.R., Hem V., Katz K.S., Ovetsky M., Wallin C., Ermolaeva O., Tolstoy I., Tatusova T., Pruitt K.D., Maglott D.R. (2015). Gene: A gene-centered information resource at NCBI. Nucleic Acids Res..

[53] Gene Ontology Consortium (2015). Gene Ontology Consortium: Going forward. Nucleic Acids Res..

[54] Kent W.J., Sugnet C.W., Furey T.S., Roskin K.M., Pringle T.H., Zahler A.M., Haussler D. (2002). The human genome browser at UCSC. Genome Res..

[55] Vawter M.P., Evans S., Choudary P., Tomita H., Meador-Woodruff J., Molnar M., Li J., Lopez J.F., Myers R., Cox D. (2004). Gender-specific gene expression in post-mortem human brain: Localization to sex chromosomes. Neuropsychopharmacology.

[56] Levine R.L., Moskovitz J., Stadtman E.R. (2000). Oxidation of methionine in proteins: Roles in antioxidant defense and cellular regulation. IUBMB Life.

[57] Kuschel L., Hansel A., Schönherr R., Weissbach H., Brot N., Hoshi T., Heinemann S.H. (1999). Molecular cloning and functional expression of a human peptide methionine sulfoxide reductase (hMsrA). FEBS Lett..

[58] Onwuli D.O., Beltran-Alvarez P. (2016). An update on transcriptional and post-translational regulation of brain voltage-gated sodium channels. Amino Acids.

[59] Leterrier C., Brachet A., Fache M.P., Dargent B. (2010). Voltage-gated sodium channel organization in neurons: Protein interactions and trafficking pathways. Neurosci. Lett..

[60] Kim B., Kang S., Kim S.J. (2012). Differential promoter methylation and histone modification contribute to the brain specific expression of the mouse Mbu-1 gene. Mol. Cells.

[61] Blanco S., Dietmann S., Flores J.V., Hussain S., Kutter C., Humphreys P., Lukk M., Lombard P., Treps L., Popis M. (2014). Aberrant methylation of tRNAs links cellular stress to neuro-developmental disorders. EMBO J..

[62] Qureshi I.A., Mehler M.F. (2010). Genetic and epigenetic underpinnings of sex differences in the brain and in neurological and psychiatric disease susceptibility. Prog. Brain Res..

[63] Luchetti S., Huitinga I., Swaab D.F. (2011). Neurosteroid and GABA-A receptor alterations in Alzheimer’s disease, Parkinson’s disease and multiple sclerosis. Neuroscience.

[64] Hillman R., Sinani J., Pendleton R. (2012). The role of the GABA_B_ receptor and calcium channels in a *Drosophila* model of Parkinson’s disease. Neurosci. Lett..

[65] Paris-Robidas S., Brochu E., Sintes M., Emond V., Bousquet M., Vandal M., Pilote M., Tremblay C., Di Paolo T., Rajput A.H. (2012). Defective dentate nucleus GABA receptors in essential tremor. Brain.

[66] Hansen T.B., Wiklund E.D., Bramsen J.B., Villadsen S.B., Statham A.L., Clark S.J., Kjems J. (2011). miRNA-dependent gene silencing involving Ago2-mediated cleavage of a circular antisense RNA. EMBO J..

[67] Kumar L., Haque R., Baghel T., Nazir A. (2017). Circular RNAs: the emerging class of non-coding RNAs and their potential role in human neurodegenerative diseases. Mol. Neurobiol..

[68] McGeer P.L., McGeer E.G. (2004). Inflammation and neurodegeneration in Parkinson’s disease. Parkinsonism Relat. Disord..

[69] López González I., Garcia-Esparcia P., Llorens F., Ferrer I. (2016). Genetic and transcriptomic profiles of inflammation in neurodegenerative diseases: Alzheimer, Parkinson, Creutzfeldt–Jakob and Tauopathies. Int. J. Mol. Sci..

[70] Yan J., Fu Q., Cheng L., Zhai M., Wu W., Huang L., Du G. (2014). Inflammatory response in Parkinson’s disease (Review). Mol. Med. Rep..

[71] Lindqvist D., Hall S., Surova Y., Nielsen H.M., Janelidze S., Brundin L., Hansson O. (2013). Cerebrospinal fluid inflammatory markers in Parkinson’s disease—Associations with depression, fatigue, and cognitive impairment. Brain Behav. Immun..

[72] Bekhbat M., Neigh G.N. (2018). Sex differences in the neuro-immune consequences of stress: Focus on depression and anxiety. Brain Behav. Immun..

[73] International Parkinson’s Disease Genomics Consortium (IPDGC), Wellcome Trust Case Control Consortium 2 (WTCCC2) (2011). A two-stage meta-analysis identifies several new loci for Parkinson’s disease. PLoS Genet..

[74] Nalls M.A., Pankratz N., Lill C.M., Do C.B., Hernandez D.G., Saad M., DeStefano A.L., Kara E., Bras J., Sharma M. (2014). Large-scale meta-analysis of genome-wide association data identifies six new risk loci for Parkinson’s disease. Nat. Genet..

[75] Qureshi I.A., Mehler M.F. (2012). Emerging roles of non-coding RNAs in brain evolution, development, plasticity and disease. Nat. Rev. Neurosci..

[76] Ravindran K., Mark R.C. (2015). Pathways to Parkinsonism Redux: Convergent pathobiological mechanisms in genetics of Parkinson’s disease. Hum. Mol. Genet..

[77] Chang D., Nalls M.A., Hallgrímsdóttir I.B., Hunkapiller J., van der Brug M., Cai F., Kerchner G.A., Ayalon G., International Parkinson’s Disease Genomics Consortium, 23andMe Research Team (2017). A meta-analysis of genome-wide association studies identifies 17 new Parkinson’s disease risk loci. Nat. Genet..

